# Advances and challenges in plant molecular marker technologies and their applications in the artificial intelligence empowered era

**DOI:** 10.3389/fpls.2025.1757949

**Published:** 2026-01-15

**Authors:** Xiaoxu Li, Zhengrong Hu, Wen Yu, He Xie, Xuebo Wang, Pingjun Huang, Xinyao Zhang, Jiashuo Yang, Yangyang Li, Weicai Zhao, Xiaonian Yang, Zhenchen Zhang, Wenxuan Pu, Zhiyuan Li

**Affiliations:** 1 Technology Center, China Tobacco Hunan Industrial Co., Ltd., Changsha, China; 2 Beijing Life Science Academy, Beijing, China; 3 Tobacco Research Institute, Chinese Academy of Agricultural Sciences, Qingdao, China; 4 Hunan Tobacco Science Research Institute, Changsha, China; 5 Institute of Tobacco Science, Fujian Provincial Tobacco Company, Fuzhou, China; 6 Yunnan Academy of Tobacco Agricultural Sciences, Kunming, China; 7 Tobacco Science Research Institute of Guangdong Province, Shaoguan, China; 8 Crops Research Institute, Guangdong Academy of Agricultural Sciences, Guangzhou, China

**Keywords:** artificial intelligence, KASP, mGPS, plant molecular markers, SNP genotyping

## Abstract

Plant molecular marker technologies have reshaped crop genetics and breeding by making it possible to analyse genome-wide variation with a precision that phenotype-based selection, even in experienced programmes, cannot reach in routine practice. This review summarises recent progress in marker platforms from classical RFLP and SSR systems to high-throughput SNP genotyping, with emphasis on KASP, multiple nucleotide polymorphism and multi-gene panel technologies, and on sequencing-based methods such as GBS, GBTS and Hyper-seq that often serve as an upstream discovery layer for targeted assays and databases. These platforms are increasingly integrated into practical workflows for marker-assisted and genomic selection, DNA fingerprinting, germplasm characterisation and plant variety protection, and multi-locus markers have become a central tool for high-resolution DUS testing and EDV determination that adds an independent layer of evidence to morphology-based assessments. Key challenges now include cross-platform standardisation, design of marker panels that balance cost with information content, interoperability of databases across institutions and countries, and the definition of molecular distance thresholds that are acceptable both biologically and in legal and regulatory settings. The review also considers the rapid integration of molecular marker data with artificial intelligence, including AI-driven marker discovery and panel optimisation, genomic prediction in multi-environment trials and the concept of an intelligent seed-industry operating system that links genotypic, phenotypic and environmental information in a coherent data framework. These developments collectively point to a shift from isolated marker assays towards platform-level, AI-supported infrastructures that can accelerate variety innovation and contribute to the modernisation and quality improvement of the seed industry.

## 1 Introduction

Global food security, climate change and growing competition in the seed industry place strong pressure on plant breeding to deliver high-yielding, agronomically stable, high-quality and stress-resilient varieties within limited timeframes under economic and regulatory constraints. Traditional breeding relies heavily on visible traits and breeder experience, whereas molecular marker technologies reveal genetic variation directly at the DNA level and increase both the accuracy and efficiency of selection, so they have become central tools in modern genetic improvement programmes (Semagn et al., 2014; Lawrie and Massey, 2023; Sun et al., 2024). Plant variety protection (PVP) frameworks in many countries now require quantitative and objective assessment of distinctness, uniformity and stability (DUS), and classical DUS testing based on morphology is sensitive to environmental effects and human judgement. DNA fingerprinting and other marker systems offer more stable and reproducible support for variety identity, infringement analysis and the identification of essentially derived varieties (EDVs) (Euler et al., 2022; Yu and Chung, 2021; Annicchiarico et al., 2025).

DNA-based markers have been used in plant science since the 1980s and have accelerated genetic research and crop improvement by allowing breeders to locate genetic factors for target traits at the genotype level, which is difficult to achieve reliably using field plots alone. High-throughput sequencing in the 21st century has made it feasible to survey large numbers of loci across entire genomes and to link genomic variation to phenotypes at scales that earlier breeding programmes could not reach. Single nucleotide polymorphisms (SNPs) are now the dominant marker type because they are abundant, stable and compatible with automated platforms. In recent years, high-throughput sequencing and computing have matured, high-density SNP platforms have become widely usable in breeding, PCR-based systems such as KASP have moved from research tools to routine platforms in breeding and PVP practice, and new multi-locus marker formats such as MNPs and mGPS panels have shown strong power to distinguish closely related varieties and to support EDV analysis (Fang et al., 2021; Liu et al., 2023, Liu et al., 2024, Liu et al., 2024; Zhao et al., 2025). The continuous advance of big data and artificial intelligence (AI) has given rise to the concept of smart breeding, in which molecular markers are viewed as important components of a broader genomic feature space. In this framework, predictive models integrate genomic, environmental and phenotypic information rather than treating each data source in isolation (Xu et al., 2022; Sun et al., 2024).

This review focuses on the marker technology platform dimension, summarises advances in KASP, MNP/mGPS and related systems, describes applications in breeding, PVP and germplasm management, and outlines how marker platforms are being combined with AI to support plant molecular biology and seed-industry innovation.

## 2 Overview of advances in molecular marker technology platforms

Early marker systems such as restriction fragment length polymorphism (RFLP), random amplified polymorphic DNA (RAPD) and simple sequence repeats (SSR) were used to build genetic linkage maps, map QTLs and study genetic diversity. These systems have limitations: throughput is low, the cost per data point is high and polymorphism can be limited in some species and populations. As datasets have expanded, these constraints have become more obvious. Over the past decade, next-generation sequencing (NGS) has driven a move towards high-throughput SNP markers that now represent the mainstream in plant genetics and breeding, although classical markers remain in use in some specialised contexts where existing infrastructure or long-term datasets favour them.

### 2.1 From classical markers to high-throughput SNP platforms

Classical markers such as RFLP, RAPD, AFLP and SSR were crucial in early crop genetic research but are difficult to scale and standardise and often show limited automation and poor comparability between laboratories (Semagn et al., 2014). With the development of NGS, SNP markers distributed across genomes and easy to detect at high throughput have become the main marker type, and multiple SNP genotyping platforms have been established for different crops and applications (Lawrie and Massey, 2023; Panahi and Jalaly, 2024; Sahoo et al., 2025). Recent methodological reviews group plant SNP platforms into three broad types. PCR-based systems such as KASP, TaqMan, ARMS-PCR and HRM use allele-specific primers with fluorescent labels for precise genotyping at one or a few loci and are suitable when sample numbers are high but the number of loci is limited. Array-based SNP chips use oligonucleotide arrays to assay thousands to hundreds of thousands of fixed SNPs and are used for genome-wide scans, population studies and identification of core markers. Sequencing-based genotyping methods including Genotyping by Sequencing (GBS), Genotyping by Target Sequencing (GBTS), RAD-seq and target-capture schemes produce SNP data by sequencing reduced-representation libraries or defined genomic regions. Falling sequencing costs have made sequencing-based genotyping central to variant discovery and high-dimensional genomic analysis, while PCR- and array-based methods are often used for routine marker-assisted selection, fingerprinting and quality control (Panahi and Jalaly, 2024; Zhang et al., 2025). Many crops now follow a general pattern in which sequencing-based discovery is used to design marker panels and these panels are then implemented on KASP or MNP platforms and linked to breeding and PVP databases (Shen et al., 2021; Yang et al., 2024; Zhang et al., 2025).

### 2.2 KASP: from single-locus genotyping to platform-level deployment

KASP (Kompetitive Allele Specific PCR) is a competitive allele-specific PCR method that has become a major single-locus SNP genotyping platform in crops and horticultural species. It works on standard real-time PCR instruments, uses a simple reaction setup and is relatively low cost, which makes it suitable for large breeding and testing programmes in both public and private sectors (Semagn et al., 2014; Sahoo et al., 2025). A typical KASP assay uses two allele-specific primers with different fluorescent tails and one common primer, the allele-specific primers compete for binding to the target template and genotypes are called from the fluorescence patterns (Semagn et al., 2014). KASP now functions as a broader platform for breeding and variety monitoring. It allows efficient conversion of SNPs discovered by GBS or resequencing into targeted markers, supports flexible assembly of crop and trait specific KASP panels and can be standardised across laboratories to support quality control in large breeding organisations (Lawrie and Massey, 2023; Sahoo et al., 2025; Sun et al., 2024). The method is widely used in wheat, rice, maize, soybean, pea and other crops for genotyping key loci, building linkage maps and supporting genotype identification, purity testing and marker-assisted selection. KASP has also been introduced into medicinal or dual-use crops. Yang et al. (2024) used whole-genome data from sweet potato to select core SNPs and built a KASP marker system for diversity analysis, population structure and DNA fingerprints that supports variety identification and breeding. Shen et al. (2021) used GBTS to select representative SNPs, converted them to KASP markers and built a fingerprint database of 372 broccoli accessions, demonstrating a complete pipeline from sequencing-based discovery to KASP implementation. These and similar studies show that KASP is both a cost-effective genotyping tool and an important link between genome-wide variant discovery and breeding and PVP applications (Semagn et al., 2014; Lawrie and Massey, 2023; Sahoo et al., 2025; Yang et al., 2024; Shen et al., 2021, Shen et al., 2025).

### 2.3 Multi-locus marker systems: MNP and mGPS

In PVP and in the discrimination of closely related varieties a single SNP or SSR locus often does not provide enough information, especially for near-isogenic lines or clonally propagated varieties. Multiple nucleotide polymorphism (MNP) markers refer to short haplotypes composed of several tightly linked SNPs identified through sequencing, and increase the information content per marker. Fang et al. (2021) proposed MNP-Seq, in which short genomic fragments containing several SNPs are sequenced as a unit so that several allelic differences are captured in one read and the polymorphic information per marker is increased. MNP markers have since been tested in different organisms. Liu et al. (2023) developed an MNP-based identification system for *Flammulina filiformis* cultivars. Liu et al. (2024) established an MNP-based identification system for grapevine cultivars for variety authentication and germplasm management. Liu et al. (2024) showed that MNP markers can distinguish closely related chrysanthemum varieties and clonal lines and can therefore support high-resolution EDV assessment. mGPS (multi-gene panel SNP) denotes a multiplexed, targeted sequencing panel that integrates multiple loci and using multiplex PCR and reduced-representation sequencing to genotype many SNPs in parallel. These panels lower sequencing cost while retaining coverage of key genomic regions and are useful for building medium-density marker sets for fingerprinting and marker-assisted selection (Panahi and Jalaly, 2024; Sun et al., 2024). Taken together, MNP and mGPS systems reflect a transition from single-locus markers to multi-locus configurations with enhanced information content and are especially well suited for plant variety protection and high-resolution germplasm discrimination in contexts requiring fine-scale differentiation (Liu et al., 2024, Liu et al., 2024; Zhao et al., 2025).

### 2.4 Upgrading and integrating sequencing and array platforms

NGS-based methods such as GBS, GBTS and Hyper-seq increasingly replace or supplement SNP chips for high-dimensional genomic studies. These approaches are now used to analyse genetic diversity, construct high-density linkage or association maps and identify core SNP sets (Panahi and Jalaly, 2024; Sun et al., 2024). Zhang et al. (2025) applied Hyper-seq to Chinese chive germplasm, carried out population genomic analysis, built a core collection and created an SNP fingerprint database, which illustrates an efficient route from sequencing to fingerprints. In many crops, GBS datasets have been mined to select SNPs that can be converted into KASP or MNP markers and used in downstream, application-oriented platforms (Yang et al., 2024; Shen et al., 2021). These developments point to a three-layer structure for current marker technology: a discovery layer based on sequencing that records genomic variation; an application layer using platforms such as KASP, MNP and mGPS for routine genotyping; and a database and decision layer that stores fingerprints and breeding data and supports data-driven decisions (Sun et al., 2024; Xu et al., 2022; Zhang et al., 2025).

## 3 Applications of molecular marker technologies

Marker-assisted selection and backcrossing, now often implemented through multi-locus KASP and panel-based genotyping, have evolved into platform workflows that also support genomic selection, where genome-wide SNP data and AI-enabled models improve prediction accuracy and accelerate multi-trait, multi-environment breeding. At the same time, multi-locus systems including MNP and mGPS provide both functional and identity information, allowing the same marker sets to serve breeding needs and plant variety protection. In PVP, DNA fingerprinting and these high-resolution marker panels now complement classical, morphology-based DUS testing and are increasingly used as quasi-evidentiary tools for EDV determination and variety disputes, without yet fully replacing phenotypic criteria.

### 3.1 Breeding applications: from MAS to smart breeding

In crop improvement, marker-assisted selection (MAS) and marker-assisted backcrossing (MABC) remain core applications. These rely on markers closely linked to genes or QTLs that influence target traits and help breeders select individuals in early generations, for recessive traits or under conditions where phenotypes are noisy or slow to measure (Semagn et al., 2014; Sun et al., 2024). With the spread of KASP and panel-based genotyping, MAS now frequently uses multiple loci and platform-based workflows. In sweetpotato, Yang et al. (2024) developed a KASP system that genotypes several SNPs associated with quality and resistance traits at the same time and supports multi-trait improvement. In broccoli and *Cymbidium ensifolium*, KASP panels are used to score several trait loci and variety fingerprints in one run and to connect breeding selection with germplasm management and market supervision (Shen et al., 2021, Shen et al., 2025). For quantitative traits and traits with strong genotype-by-environment interactions, genomic selection (GS) and AI-based models are increasingly used. GS uses genome-wide SNP data from chips or sequencing as input features for GBLUP or machine learning models that predict breeding values and shorten breeding cycles while improving prediction accuracy (Sun et al., 2024; Xu et al., 2022). Xu et al. (2022) proposed an integrated genomic-enviromic prediction (iGEP) framework that adds environmental covariates to genomic prediction models and showed that including environmental data improves prediction across environments and demonstrates the value of combining marker data with AI. Multi-locus markers such as MNP and mGPS can be used in panels that capture both functional and fingerprinting information so that the same marker set can support MAS or GS and also preserve identity information relevant to PVP and variety registration (Fang et al., 2021; Yuan et al., 2022; Sun et al., 2024).

### 3.2 Plant variety protection: molecular evidence in DUS and EDV contexts

Classical DUS testing in PVP relies on morphological traits. Many studies show that morphology alone often fails to distinguish closely related varieties and is affected by environment and subjectivity (Euler et al., 2022; Yu and Chung, 2021). DNA fingerprinting has therefore become an important complementary method. Euler et al. (2022) reviewed 23 field studies that used DNA fingerprints for varietal identification and found a 20-50% mismatch between farmer-reported variety names and DNA-based identities, with even higher discrepancies in some countries. This indicates that self-reported names and morphological inspection can lead to substantial error in variety deployment and PVP. Yu and Chung (2021) surveyed PVP practice worldwide and reported that, although the UPOV Convention defines DUS in phenotypic terms, several countries including China, EU members and the USA now use DNA fingerprints in examination and enforcement as technical support or secondary evidence. In rice, Yuan et al. (2022) built a nucleotide polymorphism-based system that can distinguish EDVs from non-EDVs and thus shows how multi-locus markers can support EDV decisions. In outcrossing forage crops such as alfalfa, Annicchiarico et al. (2025) reported that combined SSR and SNP markers increase variety distinctness and improve the reliability of DUS testing and proposed a framework that uses molecular distance to support DUS decisions. In grapevine and chrysanthemum, MNP markers can distinguish clones and closely related varieties and therefore provide high-resolution tools for EDV analysis (Liu et al., 2024, Liu et al., 2024; Zhao et al., 2025). Molecular markers have thus shifted from purely technical support to a quasi-evidentiary role in PVP. They do not yet replace phenotypic DUS in most legal systems but are widely accepted as important evidence in infringement and variety dispute cases (Yu and Chung, 2021; Euler et al., 2022; Yuan et al., 2022; Annicchiarico et al., 2025).

## 4 Integration of molecular markers and AI: prospects and applications

**Figure 1:**
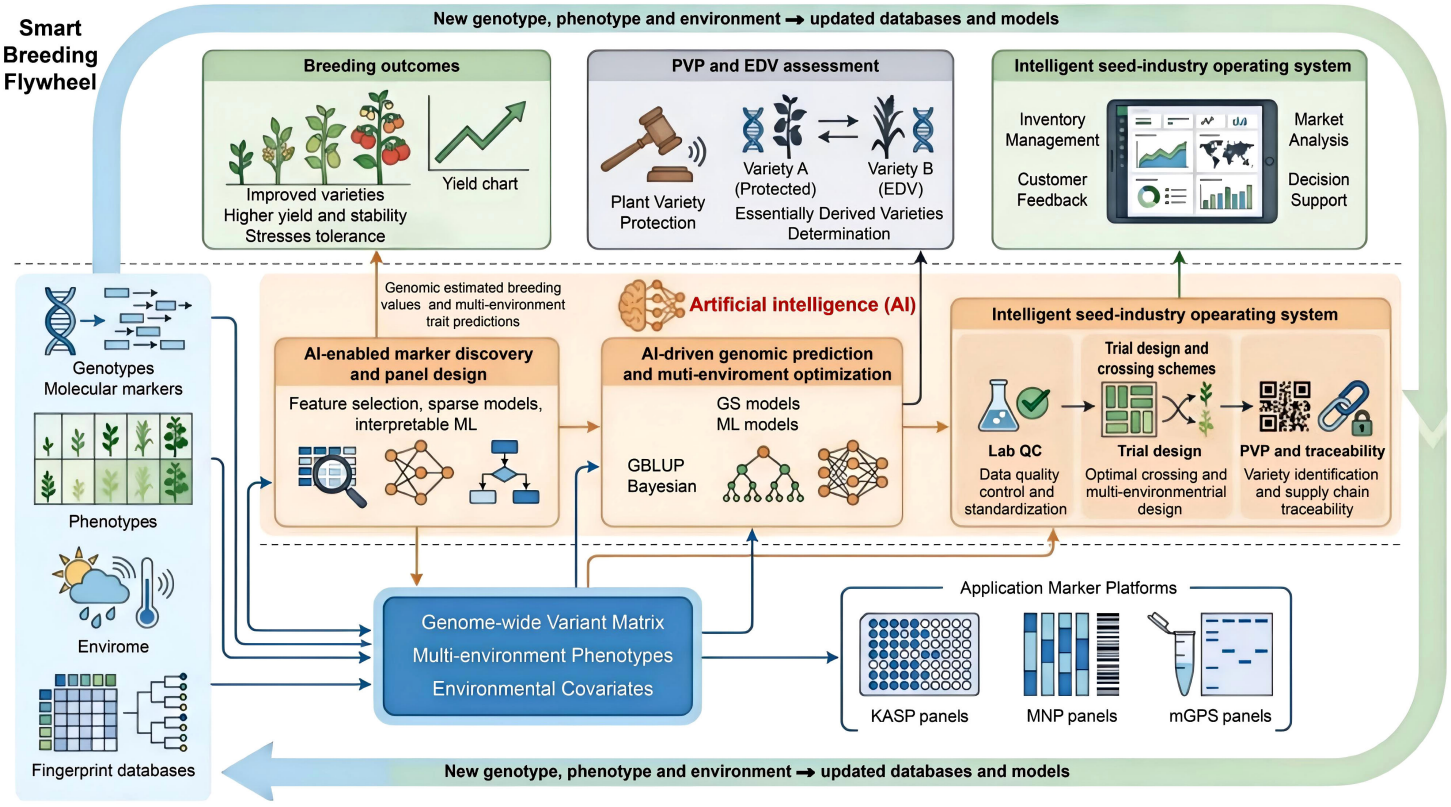
Integration of molecular marker platforms and artificial intelligence in plant breeding.

The platform-level integration of multiple molecular marker systems and scales genotypic data generation, thereby providing high-quality, structured datasets that are directly compatible with AI and machine-learning models used in genomic prediction and smart breeding frameworks. Integration of molecular marker technologies with AI is expected to change plant breeding practice (**Figure 1**). Genomics, big data and computing have converged, and concepts such as smart breeding and digital breeding reflect the use of AI to identify complex patterns in large genotype and phenotype datasets and to guide breeding decisions and genetic gain. As genomic, phenomic and environmental datasets grow, AI methods provide tools to make fuller use of marker data. Xu et al. (2022) described smart breeding as a shift from experience-based and isolated decisions to systematic procedures based on multi-source data and prediction in an AI-supported environment.

### 4.1 AI-enabled marker discovery and panel design

AI can support the design of better marker panels as well as the analysis of existing data. Genome-wide variant datasets can be analysed using feature selection, sparse models and interpretable machine learning to identify SNP and MNP loci that show stable polymorphism across populations and environments and that have strong effects on traits. These loci can then be converted into KASP or MNP assays. This approach can lower the number of markers in a panel while maintaining prediction performance and can therefore reduce genotyping costs (Lawrie and Massey, 2023; Yang et al., 2024; Fang et al., 2021). In PVP settings, clustering and metric-learning methods applied to large DUS and fingerprint datasets can help define molecular distance thresholds and identify loci with high discriminatory power. Work in this direction is already underway in MNP and Hyper-seq fingerprint systems and could support EDV threshold setting and harmonisation of variety databases across regions (Yuan et al., 2022; Liu et al., 2024, Liu et al., 2024; Zhang et al., 2025).

### 4.2 AI-driven genomic prediction and multi-environment optimization

In GS, SNP markers from KASP panels or GBS datasets are used as inputs to GBLUP, Bayesian or machine learning models that predict breeding values (Xu et al., 2022; Sun et al., 2024). Recent studies show that random forests, gradient boosting and deep neural networks can outperform linear models for multi-environment and multi-trait datasets because they can capture nonlinear relationships (Sun et al., 2024; Xu et al., 2022). As these methods are adopted, the role of marker platforms shifts from simple marker–trait associations to large feature sets that describe genome–trait–environment combinations and from tracking single genes to providing high-dimensional genomic inputs for AI models. Xu et al. (2022) showed that adding environmental variables to genomic prediction models improves accuracy across environments and is therefore important for breeding varieties adapted to broad or specific conditions under climate change.

### 4.3 Towards an intelligent seed industry operating system

Integration of markers and AI also affects how seed-industry workflows are organised. In laboratories, image recognition and anomaly detection can be used to automate quality control of KASP and GBS genotyping by interpreting fluorescence plots and read-quality statistics and can reduce manual interpretation and increase throughput. In trial design, Bayesian optimisation and reinforcement learning can use historical genotype–phenotype data to suggest crossing schemes and field designs and can improve how experimental resources are allocated (Xu et al., 2022; Sun et al., 2024). At industry and regulatory levels, DNA fingerprint databases can be linked with blockchain-based traceability systems to support reliable variety identity and origin labels, while AI tools can monitor these systems for unusual patterns and potential risk signals (Euler et al., 2022; Yu and Chung, 2021). These developments together suggest that AI can help overcome limits of human intuition, derive robust rules from large datasets and support more predictive breeding design. A likely future scenario is one in which breeders define targets and AI systems use marker and environmental databases to propose parental combinations, crossing designs and sample sizes, with models updated after each breeding cycle to gradually improve performance. Such a system will require high-quality data, interpretable models and training of researchers who understand both biology and AI. The convergence of molecular markers and AI is therefore expected to drive further innovation in breeding, and marker platforms are likely to function less as isolated wet-lab modules and more as core parts of an intelligent seed-industry operating system that integrates phenotypic, environmental and management data and supports decisions from variety creation to market deployment.

## 5 Conclusion

Plant molecular marker platforms have changed rapidly over the past decade, moving from classical SSR and RFLP markers to SNP-based high-throughput genotyping and to multi-layer, scenario-specific platforms such as KASP, MNP and mGPS. KASP platforms combine flexibility with favourable cost–performance and are now used widely in breeding, germplasm management and PVP practice. MNP and mGPS systems offer higher information density and better resolution for distinguishing closely related varieties and supporting EDV decisions. Molecular markers now form an integral part of breeding workflows, support the shift from single gene MAS to genome-wide and multi-environment prediction, and in PVP they provide key technical support for DUS testing and EDV assessment. In germplasm management and supply chain regulation, marker systems underpin the maintenance of genetic diversity, the construction of core collections and the verification of variety identity and traceability. As platforms, data systems and regulatory frameworks develop further, molecular marker technologies are likely to play an even stronger role in supporting crop variety innovation and the high-quality growth of the seed industry in an AI-enabled era.
